# Barriers to Effective Diabetes Mellitus Self-Management (DMSM) Practice for Glycemic Uncontrolled Type 2 Diabetes Mellitus (T2DM): A Socio Cultural Context of Indonesian Communities in West Sulawesi

**DOI:** 10.3390/ejihpe10010020

**Published:** 2019-11-26

**Authors:** Rian Adi Pamungkas, Kanittha Chamroonsawasdi, Paranee Vatanasomboon, Phitaya Charupoonphol

**Affiliations:** 1 Doctor of Public Health Program, Department of Family Health, Faculty of Public Health, Mahidol University, Bangkok 10400, Thailand; rian.adi@esaunggul.ac.id; 2 Department of Family Health, Faculty of Public Health, Mahidol University, Bangkok 10400, Thailand; phitaya.cha@mahidol.ac.th; 3 Department of Health Promotion and Behavioral Science, Faculty of Public Health, Mahidol University, Bangkok 10400, Thailand; paranee.vat@mahidol.ac.th

**Keywords:** uncontrolled type 2 diabetes mellitus, self-management, barriers, Indonesian communities

## Abstract

Diabetes mellitus self-management (DMSM) is an essential strategy used to maintain blood glucose levels and to prevent severe complications. Several barriers have been documented while implementing DMSM practices. A qualitative study aimed to explore barriers to effective DMSM practice among uncontrolled glycemic type 2 diabetes mellitus (T2DM) patients in Indonesia. We conducted in-depth interviews and focus group discussions (FGDs) among 28 key informants, including patients, family members, healthcare providers (HCPs), and village health volunteers (VHVs). The interviews and FGDs were audiotaped and transcribed verbatim. The results revealed six core themes with sub-categories of themes used by all participants to describe the barriers to effective DMSM practice among uncontrolled T2DM patients. The critical barriers of DMSM practice included low perception of susceptibility to and severity of the illness; inadequate knowledge and skill of diabetes mellitus self-management; lack of motivation to perform diabetes mellitus self-management; insufficient human resources; lack of social engagement; and social exclusion and feelings of embarrassment. Our findings provide valuable information regarding the barriers while implementing the DMSM practice. Healthcare providers should negotiate with both T2DM patients and caretakers to participate in a DMSM program at a community health care level.

## 1. Introduction

Type 2 diabetes mellitus (T2DM) is a rapid growing health issue, which affects approximately 415 million people worldwide [1]. The rate of T2DM in Indonesia in 2013 was about 13% of the total population, and it has increased year by year [2]. An increasing number of uncontrolled T2DM patients could impact patients, healthcare systems, and the broader economy by imposing an economic burden of medical costs and a loss of productivity [3]. Therefore, controlling blood glucose levels is crucial. 

Diabetes management is complex because T2DM patients must be able to reconcile their resources, values, and preferences towards a healthy diet, participation in physical activity, abstinence from alcohol consumption, medical adherence, blood glucose monitoring, as well as prevention of diabetes complications [4]. Self-management continuity is particularly important in diabetes care, since uncontrolled T2DM patients require optimal coordination and communication between themselves, their families, healthcare providers, and other stakeholders [5,6].

The American Diabetes Association has suggested that diabetes mellitus self-management (DMSM) practice is greatly influenced by the level of patients’ own participation in care [7]. Some studies also confirmed that DMSM practice could improve patients’ perceived obstacles to behavioral adherence [8,9], which is essential to achieving optimal glycemic control and medication adjustment in the context of daily living [10]. However, it is a challenge to perform DMSM for the persistent control of blood glucose levels. 

Several studies related to inconsistent self-management behaviors have been documented from different social contexts surrounding the patients, including family members (spouse and relatives), peers, romantic partners, and healthcare providers. It was found that different social contexts that influenced DMSM practice consisted of warmth, collaboration, and acceptance, as well as involvement in daily activities [11]. The main barriers to treatment found among patients were insufficient information on diabetes self-management [12,13], poor medical adherence [13,14], competing daily demands, emotional distress, low self-commitment [15], and insufficient social support [16]. Barriers related to healthcare providers (HCPs) included insufficient communication between HCPs and patients [17], unclear descriptions of professionals’ responsibilities within teams [13], and the ignoring of the responsibilities of fellow professionals [12]. Additional barriers of patient-related facilitators that were found included lack of motivation [18], lack of appropriate peer influence [14], lack of confidence in self-management skills, and lack of effective communication [19]. Therefore, the fundamental goal to understand diabetic self-management and glycemic control needs to be explained by several key factors, as mentioned above, particularly among different social contexts in Indonesian communities. 

## 2. The Indonesian Context on DMSM Practice

In the Indonesian context with respect to DMSM, traditional practices are closely related to the local context of people within the country. Every region has its distinct conventional customs, which make the regions unique. Their beliefs are usually displayed in the traditional practices that they carry out in society. Indonesian people within the family have traditional ways to follow their family lifestyle, and almost all extended families have their meals together with the same menu at home. On some occasions, such as on wedding ceremonies, people often gather together to cook and to eat different varieties of food. However, this situation also becomes challenging among diabetic people who must avoid certain foods, such as sweet foods and foods rich in unsaturated and trans-fats, which may affect the risk of cardiovascular diseases among T2DM patients. 

Diabetes management has mainly occurred within the community health center in the form of primary health care services. These are basic services focused on diabetes self-management beyond the *Prolanis* Program. It functions as as a health education and counselling service as well as a routine service for monthly blood glucose check-up from the district hospital to provide an outreach service at the community health center. The initial aim of the outreach service was to provide optimal benefits in terms of reduced waiting time and more time spent on counseling and health education. However, several problems were documented during program implementation. Better understanding of DMSM barriers is crucial in order to prioritize targets of intervention, and further investigation is needed to understand the obstacles and how these barriers influenced the implementation of the diabetes management program at the community health center in Indonesia.

## 3. Study Objective

We conducted a qualitative study using in-depth interviews and focus group discussions (FGDs) to identify the DMSM barriers that may indicate ineffective implementation of DMSM practice in the community context. The information was drawn from the perspective of patients, their families, healthcare providers (HCPs), and village health volunteers (VHVs). A chronic care model (CCM) was applied to determine and optimize the provision of care for patients with chronic conditions like diabetes mellitus [20]. This model also showed the interconnection between community resources, including; (1) decisions in self-management support; (2) family involvement in chronic care; (3) community resources; and (4) health service delivery, to explain how chronic patients comply with treatment and cope with their illness. A better understanding of the barriers of DMSM practice from different viewpoints among patients, their families, HCPs, and VHVs could clarify ways to help HCPs to design effective interventions for uncontrolled T2DM, especially in the Indonesian context, in order to maintain the quality of life among chronic illness patients as well as to reduce the service burden in the near future. 

## 4. Methods

### 4.1. Study Design and Setting

A qualitative study was conducted by both in-depth interviews and FGDs between 1 January and 30 February 2019. The study took place at community health centers and basic health facilities in Polewali Mandar district, West Sulawesi Province, Indonesia. This district is in a sub-urban area with a crowded population. Each participant was explained the purpose of the study and asked to sign the informed consent form to allow tape recording during the in-depth interview and FGD process. 

### 4.2. Population and Samples

The study population was comprised of those who were residing in the community of West Sulawesi, Indonesia, and who were directly involved in sharing their experiences and viewpoints on DMSM and its barriers, the DMSE program, and the context of DMSM practices among Indonesian communities. The four main groups of the study population were glycemic uncontrolled patients, caretakers responsible for taking care of T2DM patients, HCPs, and VHVs. In general, among Indonesian communities, HCPs and VHVs have close relationships with community members, since almost all of them live within the same communities and some of them are relatives of T2DM patients. 

Samples included 28 key informants purposively selected based on the inclusion criteria as follows: The first group comprised eight uncontrolled T2DM patients who had resided in the community of south Sulawesi for at least one year and were willing to participate in this study. Patients with HbA1c ≥ 6.5% and age ≥ 35 years old were also included. The second group included eight caretakers who were mainly involved in supporting T2DM patients on self-management, and who were living together with the patients. Those caretakers who had no experience in taking care of patients and who lived separately from the patients were excluded from the study. The third group comprised six HCPs and six VHVs who were involved in health services provision for T2DM patients, including routine health check-ups, blood glucose monitoring, health education, home visits, and follow-ups. 

### 4.3. Data Collection Procedure

The in-depth interviews and FGDs were conducted by using a semi-structured interview, with open-ended questions following an interview guideline, based on the main themes and sub-themes, to explore barriers to effective DMSM practice among T2DM uncontrolled patients. Each interview took place at a community health center for approximately 60 to 90 minutes. All interviews were audiotape recorded and were transcribed by the interviewer and research assistant. The interview guidelines were classified into four sub-groups of key informants as follows: (1) Glycemic uncontrolled T2DM patients included the themes of demographic information, DMSM practice, barriers to DMSM practice, and perceived support from caretakers on DMSM practice; (2) family members included the themes of demographic information, family functions to support DMSM practice, and barriers to support DMSM practice; (3) HCPs included the themes of demographic information, barriers to implement the DMSM program at a community level, provision of routine services, and standard care for T2DM patients; and (4) VHVs included the themes of demographic information, and barriers to support HCPs during implementation of the DMSM program. Before research was conducted, three experts checked the interview guidelines, and pilot-testing among three uncontrolled T2DM and their family members was conducted in another community near the study site. A detailed explanation of the themes explored by each sub-group of key informants is summarized in Table 1. 

### 4.4. Data Analysis 

Data were transcribed and translated from an audio recorder. After data were collected, researchers underwent a data reduction process to identify the main themes and sub-themes. Content analysis was used to identify, analyze, and report themes within the local context. Parallel techniques to extract key themes and sub-themes were done by two researchers in order to make a consensus agreement of the findings. The final step of this qualitative data analysis was to create the categories and sub-categories of themes based on each group of key informants’ viewpoints.

## 5. Theoretical Framework 

We applied a Chronic Care Model (CCM) [21] to classify barriers to DMSM practice among patients, their families, HCPs, and VHVs. The major step of the CCM began by identifying patients’ needs and barriers to controlling the DMSM practice. This information was obtained from T2DM patients’ and caretakers’ perspectives. In terms of implementing DMSM practice, family support is a crucial component to maintain the behavioral change and blood glucose control. Chronic conditions develop slowly and may become worse over an extended period of time. Thus, family barriers need to be explored from the perspective of the caretakers. 

Chronic care management services are generally provided as Medicaid and Medicare for patients with a comprehensive care plan. HCPs are important people for delivering the medical treatment; however, some obstacles are often faced in implementing the program. Therefore, information from HCPs about barriers is needed for analysis. Care coordination in primary care practice involves deliberately organizing patient care activities among all of the people concerned with patient care to achieve effective care. VHVs are members of the community who help diabetic patients to maintain DMSM practices. Ensuring the effectiveness of program implementation in primary health care, care coordination, and its barriers is an important aspect and need to be explored from VHV perspectives. The linkage between each element in the CCM model, especially patients, caretakers, HCPs, and VHVs, guided us to clearly identify barriers and design proper interventions for chronic diseases, especially for diabetes mellitus patients.

## 6. Results

### 6.1. Demographic Characteristics

Eight uncontrolled T2DM patients were recruited and interviewed. Five of them were female with an average age of 45 (SD = 2.01) years old. Duration of diabetes ranged from two to seven years. Seven out of eight family members were female. The majority of family members had completed high school and were currently residing with the patients. Regarding DMSM practice, between men and women it was found that women preferred to eat sweet food and beverages such as cake and soft drinks more than men. By contrast, women were more concerned about medical adherence and blood glucose monitoring than men. 

Most of the HCPs were female with three of them having completed a Diploma III in nursing, while the other three completed a bachelor’s degree in nursing. The HCPs had been working in the diabetes care unit at a community health center for an average of more than four years. Six VHVs were recruited from different villages, and all VHVs were females. The VHVs had been working for four or fewer years to help HCPs to monitor diabetes patients in the communities. All of them had graduated at the high school level. 

### 6.2. Barriers to DMSM Practice 

The analysis of DMSM barriers followed the CCM model. The results were systematically presented from patients’ viewpoints, families’ viewpoints’ (obtained from caretakers), HCPs’ viewpoints (obtained from nurses), and the community’s viewpoint (obtained from VHVs). Five relevant themes were discussed to describe the barriers to DMSM practice for uncontrolled T2DM patients in primary health care.

#### 6.2.1. Low Perception of Susceptibility and Severity of Illness

Perceived susceptibility to disease has an essential role in explaining the health-related behavior of individuals. Some patients mentioned that they did not control blood glucose while living with diabetes. They did not avoid certain foods and ate what they wanted. Another patient explained that she did not follow the medication adherence because of doubts about the expected benefits and efficacy of treatments. She preferred to consume some herbal medicine from a traditional healer. Similarly, a caretaker mentioned that herbal medicine is better than diabetic drugs for maintaining blood glucose levels. 

Other patients explained that sweet foods and fast food are not so dangerous for diabetes patients. They always ate like normal people even though they were diabetes patients. This was similar with the caretakers, who mentioned that they did not have separate meals from the patients but all of them ate together at home. This was also recounted by HCPs: that some patients had difficulties in restricting unhealthy food even they knew that unhealthy food may affect their blood glucose level. Another HCP concluded that low perceptions of susceptibility and severity of the illness made patients not adhere to the medication regimens.

*“I am doubtful about the effectiveness of medicine on my health outcomes”*;

(Patient, female, 41-years-old)

*“Herbal medicine is better than the diabetes drug”*;

(Daughter, 30-years-old)

*“Low perception on severity of illness”*

(HCP 1, female, 35-years-old)

#### 6.2.2. Inadequate Knowledge and Skill of Diabetes Mellitus Self-Management 

In general, patients expressed that they were not well informed about diabetes, although some of them received information from the community health center and electronic media; but it was still unclear. Patients also had misconceptions about diabetes and its management. They thought that diabetes was caused by high carbohydrate consumption. They were not certain about what appropriate foods to eat and how to prepare healthy food for diabetes patients. Another patient mentioned that they did not perform any physical activity because they were not sure how to do proper exercise that was appropriate for their health status.

Family members’ opinions were similar in terms of inadequate information about to support T2DM patients on self-management. They still did not know how to take care of patients with diabetes mellitus. One family member assisted the patient to seek information from the internet; however, it made her more confused because of unclear and controversial information from different sources. 

During the FGD process, one VHV mentioned a lack of experience and misunderstanding about diabetes control as the main barriers of patients and their families to diabetes management, while HCPs noted some problems in utilizing the diabetes self-management education (DMSE) for diabetes patients in the community. Some of them reported that DMSE was delivered by using one way teaching rather than counseling or participatory learning by sharing experiences among T2DM patients and their families. 

Another HCP expressed the difficulty of educating patients because of lack of educational media and booklets for patients. Some HCPs mentioned that they provided education every week without any evaluation form to report on behavioral changes among the patients after receiving the DMSE.

*“Misconception of diabetes and proper exercise for diabetes”*;

(Patient, female, 41-years-old)

*“Confusing because of unclear and controversial information”*;

(Wife, 45-years-old)

*“Lack of experience and misunderstanding on diabetes care”*

(VHV 2, female, 40-years-old)

#### 6.2.3. Lack of Motivation to Perform the Diabetes Mellitus Self-Management

Patients expressed their feelings about performing physical activity. One patient indicated that she always spends her time staying at home rather than performing physical activities outside. One patient mentioned the lack of time to perform physical activity or to visit a community health center for a blood glucose check-up. In contrast, the entire families’ reasons for a lack of time did not cause all individuals to neglect their physical exercise. A main reason for not exercising was its impacts on fatigue after finishing. Almost all patients tried to avoid activities that pulled them out of their comfort zones. One participant felt uncomfortable when doing an exercise. Thus, she avoided performing daily exercise.

During the interview process, we noted that lack of a role model of DMSM was crucial as a main barrier to DMSM. The role model is typically important, particularly among those patients with a chronic illness, such as diabetes mellitus. One patient mentioned that her husband had always been a role model. He demonstrated the sharing of his feelings about an appropriate way to live with diabetes. Therefore, it was incredibly touching and inspiring personally to the patient.

Conflict within the family also became a barrier to effective DMSM practice among uncontrolled T2DM patients. In this part, some patients mentioned that the differences between the diets of family members and participants during mealtimes separated them from each other, and that family members sometimes sabotaged the patients by being strict about food, which caused some conflicts. Other patients also expressed their feelings of sadness because their relatives did not concern themselves enough with the severity of their conditions. As a result, some of them felt excluded from their family members and isolated from potential social ties. This can be seen in the patient quotes below:

*“[I prefer] Spending time at home rather than physical activity outside”*;

(Patient, female, 48-years-old)

*“[There is] Family conflict and sabotage of food at meal time”*

(Wife, female, 50-years-old)

#### 6.2.4. Insufficient Manpower 

Healthcare providers have essential roles in providing adequate health literacy regarding DMSM practices. Inadequate health literacy could impact the misconception of diabetes management. In this part of the interview, one health care provider mentioned that she did not understood diabetes care management and how to prevent diabetes complications. As a result, she only provided the patients with some leaflets which did not have clear information about diabetes management. A lack of confidence in dealing with diabetes management was also expressed by some VHVs in the community. Some of them highlighted a lack of knowledge on diabetes management and limited information to support patients in dealing with their behaviors. 

Most of the HCPs reported long and intense days spent in a community health center. Average working hours were 8 –10 hours per day. HCPs also mentioned that they needed to do administrative work and to provide treatment and care for approximately eight hours for hospitalized patients. Some of them also described the additional job of responding not only to the diabetes unit but also working on diseases prevention and control units, such as immunization, tuberculosis, and other programs, because of a limited number of HCPs. Another HCP mentioned the referral of patient activities from the community health center to the provincial hospital when patients needed more complex management. As a result, the continuing of the DMSM program sometimes became one of the barriers due to the high workload of HCPs at a community health center.

*“I have been faced with a high workload everyday by doing administrative work, providing treatment, and caring for hospitalized patients”*

(HCP 4, female, 33-year-old)

#### 6.2.5. Social Exclusion and Feelings of Embarrassment

Some VHVs mentioned that diabetic patients felt social exclusion due to victim blaming about their disease. Another VHV also expressed the social perception of diabetes regarding the excessive negative image of diabetes. Diabetes was perceived as a silent and gradual killer. Some people believed that diabetes is a long-term period of suffering that induced the sufferers towards death. 

Some patients expressed the social exclusion and feelings of embarrassment due to diabetic foot ulcers. They were not capable of joining social activities in the communities. One patient also mentioned that she felt uncomfortable and embarrassed when performing DMSM practices such as blood glucose monitoring or taking insulin in public places:

*“Not capable and low effort to join any social activity”*;

(Patient, male, 55-years-old)

*“Social perception and negative image of diabetes”*

(VHV 5, female, 28-years-old)

Table 2 show barriers to DMSM practice based on the chronic care model (CCM). Five themes were obtained from this study including (1) low perception on susceptibility and severity of diabetes, (2) inadequate knowledge and skill on diabetes mellitus self-management, (3) lack of motivation to perform the diabetes mellitus self-management, (4) insufficient manpower, and (5) social exclusion and feelings of embarrassment.

## 7. Discussion

This study found that women preferred to eat sweet food and beverages more than men, whereas men were less concerned about drug adherence and blood glucose monitoring. This finding was incongruent with a study done in Thailand, which found that men were more confident about treatment effectiveness than women [22]. Another study mentioned that men focused on blood glucose monitoring, whereas women focused on food restrictions to control their blood glucose [23]. 

Through our findings, we can identify some barriers and how those barriers undermine the efforts of patients to implement the effective DMSM practices. Using a chronic care model (CCM), researchers identified all barriers to DMSM practice among patients with T2DM, including low perceptions about susceptibility and severity of the illness, inadequate knowledge and skills about diabetes mellitus self-management, lack of motivation to perform the diabetes mellitus self-management, insufficient manpower, and social exclusion and feelings of embarrassment. The results add to the growing literature around barriers of the patients, family members, and healthcare providers to T2DM self-management [24,25,26,27,28,29]. The findings of this study also are essential for healthcare providers to help T2DM patients become more effective in addressing the common problems to DMSM practice, as well as to provide necessary information before developing a program for uncontrolled T2DM. 

Perceived severity has a vital role in explaining health-related behaviors and increasing precautionary actions. Attitudes and beliefs are crucial factors that influence perceptions about the susceptibility and severity of the illness. A similar finding in a previous study conducted in the UK mentioned that personal perception was associated with unawareness of physical exercise [30]. Another study showed the association between personal perception and attitudes toward self-care with higher scores of self-care practice [31]. One study also reported that patients’ attitudes have a vital role in patients’ willingness to adhere to diabetes management [32]. Another reason for low perception of susceptibility and severity of illness was caused by doubt about expected benefits and efficacy of treatment [33]. 

Deep understanding of diabetes management is an essential aspect and significant predictor for self-care behaviors, particularly for diabetes patients [34]. Lack of health information was associated with misconception in diabetes management. There was some evidence showing that sufficient knowledge about diabetes management was associated with a greater likelihood of performing DMSM practice [35], perceived barriers in blood glucose monitoring [36], and medication adherence [37]. In contrast, another study revealed that poor diabetes health literacy was independently associated with worse DMSM practice and glycemic control [38]. 

Although patients had experienced living with diabetes mellitus, some of them were less participatory in the DMSM program at the community health center. This was caused by a lack of motivation to perform the DMSM. Doing diabetes management such as exercise was the hardest part of the constraints perceived by the participants based on their viewpoints, including feeling too lazy to do exercise and feeling tired and uncomfortable after doing exercise. Another finding also indicated that participants were busy in daily work. This is likely to be the biggest reason for them to spend their time earning money without doing any exercise. Incongruent findings with the current study were obtained in a United Kingdom study, which revealed that T2DM patients intended to substitute exercise with household work when they stayed at home [39].

Lack of support and conflict within the family can affect DMSM practice. Similarly to what a previous study showed, appropriate support is associated with less conflict within the family, less feelings of criticism, and comfort with maintaining the DMSM practice. However, another study found insufficient support from family members, and lack of consensus agreement to make decisions on DMSM practice are challenges for controlling blood glucose levels [40]. 

One of the significant constraints toward implementation of the DMSM program in the community was the high workload of HCPs. The shortage of HCPs due to a high turnover rate and unequal distribution in the remote areas was found to be a major constraint of healthcare services in dealing with long term care in Indonesian communities, especially toward home visits for continuing care and follow-up to strengthen DMSM practice and to prevent diabetes complications. A study reported that low quality of care and employment capacities were closely associated with shortages of HCPs [41]. Another study conducted in China showed a positive correlation between availability and distribution of the health workforce and health seeking behavior among diabetes mellitus patients [42]. Thus, increasing the number of HCPs at community health centers is crucial to maintain adequate levels of healthcare services for DM patients.

Social exclusion and feelings of embarrassment are other barriers in the DMSM practice among T2DM patients. The issue is due to victim blaming about their disease, which has impacts on social exclusion and feelings of embarrassment towards DMSM practices, such as blood glucose monitoring or taking insulin in public places. A study conducted among adolescents and an adult diabetes population reported that feelings of embarrassment about disease progression were influenced by social engagement, social exclusion, and poor glycemic control [43]. Another study showed that negative perception led to a more significant burden of diabetes care and avoiding social engagement among patients in Asian countries [44]. This perspective is also associated with a disproportionate burden on diabetic patients, particularly women, and affects their marriage potential [45,46]. Therefore, HCPs, who mainly work with diabetes patients, should ask for consent and negotiate with both T2DM patients and their families before designing prevention and control strategies. The strategies may include community education, family education, and capacity building for healthcare providers as a core component in delivering diabetes programs and activities in Indonesian communities.

## 8. Conclusions

Our findings help to determine the barriers and needs of Indonesian communities regarding diabetes mellitus self-management practices. We collected information from different perspectives of patients, family members, HCPs, and VHVs. Data were obtained by both in-depth interviews and FGDs among key informants. T2DM patients revealed different components of barriers, which may have effects on the inconsistency of DMSM practices. The healthcare providers should consider how to provide support to those patients with uncontrolled T2DM and their families. Such a program intervention also needs to take into account both patients’ and families’ contexts. Thus, they may need to conduct the intervention at the community level rather than through an individual approach.

## Figures and Tables

**Table 1:** Description of sample selection and number of samples enrolled in each group.

Participants	Sample Size	Selection Criteria	Themes
Uncontrolled T2DM patients	8	HbA1c ≥ 6.5% Aged ≥ 35 years’ old Has been living at least one year with diabetes	DMSM practice Barriers to DMSM practice Perceived support from caretaker of DMSM practice
Family members	8	The main caretaker responsible for diabetes care	Family function to support DMSM practice Barriers to support DMSM practice
HCPs	6	Having a role in diabetes care in the community health centers Worked in diabetes with at least one year of experience	Barriers to implement the DMSM program at the community level Provision of routine services and standard care for T2DM patients
VHVs	6	Having a role in supporting diabetes care Having experience in diabetes care of at least one year	Barriers to support HCPs while implementing the DMSM program

**Table 2:** Barriers to DMSM practice based on the chronic care model (CCM).

Categories of Themes	Sub-Theme Categories
Low perception of susceptibility and severity of diabetes	Doubt about the expected benefits and efficacy of treatment
Inadequate knowledge and skill on diabetes mellitus self-management	Misconceptions about diabetes and its management Lack of skills for deciding proper management Limited educational media and booklets One way teaching of DMSE
Lack of motivation to perform the diabetes mellitus self-management	Lack of social support on DMSM practice Lack of time No role model Family conflict
Insufficient manpower	High workload and job demands on every project Lack of confidence and experience in dealing with diabetes management
Social exclusion and feelings of embarrassment	Social exclusion due to victim blaming of the disease Social stigma and negative image about disease progression

## References

[1] International Diabetes Federation (IDF) (2015). Diabetes Atlas.

[2] Idris H., Hasyim H., Utama F. (2017). Analysis of Diabetes Mellitus Determinants in Indonesia: A Study from the Indonesian Basic Health Research 2013. Acta Med. Indones.

[3] World Health Organization (2016). Global Report on Diabetes. https://apps.who.int/iris/bitstream/handle/10665/204871/9789241565257_eng.pdf;jsessionid=8D2436541C39EDF6A434AA55F0087A0C?sequence=1.

[4] Pamungkas R.A., Chamroonsawasdi K., Vatanasomboon P. (2017). A Systematic Review: Family Support Integrated with Diabetes Self-Management among Uncontrolled Type II Diabetes Mellitus Patients. Behav. Sci..

[5] Gulliford M.C., Naithani S., Morgan M. (2007). Continuity of care and intermediate outcomes of type 2 diabetes mellitus. Fam. Pract..

[6] Haggerty J.L., Reid R.J., Freeman G.K., Starfield B.H., Adair C.E., McKendry R. (2003). Continuity of care: A multidisciplinary review. BMJ.

[7] American Diabetes Association (ADA) (2015). Foundations of care: Education, nutrition, physical activity, smoking cessation, psychosocial care, and immunization. Diabetes Care.

[8] Vermeire E., Royen P.V., Coenen S., Wens J., Denekens J. (2003). The adherence of type 2 diabetes patients to their therapeutic regimens: A qualitative study from the patient’s perspective. Pract. Diabetes.

[9] Vermeire E., Hearnshaw H., Rätsep A., Levasseur G., Petek D., van Dam H., van der Horst F., Vinter-Repalust N., Wens J., Dale J. (2007). Obstacles to adherence in living with type-2 diabetes: An international qualitative study using meta-ethnography (EUROBSTACLE). Prim. Care Diabetes.

[10] Reyes J., Tripp-Reimer T., Parker E., Muller B., Laroche H. (2017). Factors Influencing Diabetes Self-Management among Medically Underserved Patients with Type II Diabetes. Glob. Qual. Nurs. Res..

[11] Wiebe D.J., Helgeson V., Berg C.A. (2016). The social context of managing diabetes across the life span. Am. Psychol..

[12] Wens J., Vermeire E., Royen P.V., Sabbe B., Denekens J. (2005). GPs’ perspectives of type 2 diabetes patients’ adherence to treatment: A qualitative analysis of barriers and solutions. BMC Fam. Pract..

[13] Goderis G., Borgermans L., Mathieu C., Van Den Broeke C., Hannes K., Heyrman J., Grol R. (2009). Barriers and facilitators to evidence-based care of type 2 diabetes patients: Experiences of general practitioners participating to a quality improvement program. Implement. Sci..

[14] Cochrane L.J., Olson C.A., Murray S., Dupuis M., Tooman T., Hayes S. (2007). Gaps between knowing and doing: Understanding and assessing the barriers to optimal health care. J. Contin. Educ. Health Prof..

[15] Tong W.T., Vethakkan R.S., Ng C.J. (2015). Why do some people with type 2 diabetes who are using insulin have poor glycaemic control? A qualitative study. BMJ Open.

[16] Miller T.A., DiMatteo M.R. (2013). Importance of family/social support and impact on adherence to diabetic therapy. Diabetes, metabolic syndrome, and obesity: Targets and therapy. Diabetes Metab. Syndr. Obes. Targets Ther..

[17] Kripalani S., LeFevre F., Phillips C.O., Williams M.V., Basaviah P., Baker D.W. (2007). Deficits in Communication and Information Transfer Between Hospital-Based and Primary Care Physicians: Implications for Patient Safety and Continuity of Care. JAMA.

[18] Van Bruggen R., Gorter K.J., Stolk R.P., Verhoeven R.P., Rutten G.E. (2008). Implementation of locally adapted guidelines on type 2 diabetes. Fam. Pract..

[19] Nam S., Chesla C., Stotts N.A., Kroon L., Janson S.L. (2011). Barriers to diabetes management: Patient and provider factors. Diabetes Res. Clin. Pract..

[20] Bodenheimer T., Wagner E.H., Grumbach K. (2002). Improving primary care for patients with chronic illness: The chronic care model, Part 2. JAMA.

[21] Wagner E.H., Austin B.T., Davis C., Hindmarsh M., Schaefer J., Bonomi A. (2001). Improving chronic illness care: Translating evidence into action. Health Aff. (Millwood).

[22] Boonsatean W., Carlsson A., Dychawy R.I., Ostman M. (2018). Sex-related illness perception and self-management of a Thai type 2 diabetes population: A cross-sectional descriptive design. BMC Endocr. Disord..

[23] Mathew R., Gucciardi E., De Melo M., Barata P. (2012). Self-management experiences among men and women with type 2 diabetes mellitus: A qualitative analysis. BMC Fam. Pract..

[24] Jones L., Crabb S., Turnbull D., Oxlad M. (2014). Barriers and facilitators to effective type 2 diabetes management in a rural context: A qualitative study with diabetic patients and health professionals. J. Health Psychol..

[25] Elliott D.J., Robinson E.J., Sanford M., Herrman J.W., Riesenberg L.A. (2011). Systemic barriers to diabetes management in primary care: A qualitative analysis of Delaware physicians. Am. J. Med. Qual..

[26] Campbell D.J., Manns B.J., Hemmelgarn B.R., Sanmartin C., Edwards A., King-Shier K. (2017). Understanding Financial Barriers to Care in Patients with Diabetes. Diabetes Educ..

[27] Mogre V., Johnson N.A., Tzelepis F., Paul C. (2019). Barriers to diabetic self-care: A qualitative study of patients’ and healthcare providers’ perspectives. J. Clin. Nurs..

[28] Sohal T., Sohal P., King-Shier K.M., Khan N.A. (2015). Barriers and Facilitators for Type-2 Diabetes Management in South Asians: A Systematic Review. PLoS ONE.

[29] Singh H., Cinnirella M., Bradley C. (2012). Support systems for and barriers to diabetes management in South Asians and Whites in the UK: Qualitative study of patients’ perspectives. BMJ Open.

[30] Kennedy A., Narendran P., Andrews R.C., Daley A., Greenfield S.M. (2018). Attitudes and barriers to exercise in adults with a recent diagnosis of type 1 diabetes: A qualitative study of participants in the Exercise for Type 1 Diabetes (EXTOD) study. BMJ Open.

[31] Karimy M., Koohestani H.R., Araban M. (2018). The association between attitude, self-efficacy, and social support and adherence to diabetes self-care behavior. Diabetol. Metab. Syndr..

[32] Rubin R.R. (2005). Adherence to pharmacologic therapy in patients with type 2 diabetes mellitus. Am. J. Med..

[33] Markowitz S.M., Gonzalez J.S., Wilkinson J.L., Safren S.A. (2011). A review of treating depression in diabetes: Emerging findings. Psychosomatics.

[34] Koetsenruijter J., Van Lieshout J., Lionis C., Portillo M.C., Vassilev I., Todorova E., Foss C., Gil M.S., Knutsen I.R., Angelaki A. (2015). Social Support and Health in Diabetes Patients: An Observational Study in Six European Countries in an Era of Austerity. PLoS ONE.

[35] Persell S.D., Keatin N.L., Landrum M.B., Landon B.E., Ayanian J.Z., Borbas C., Guadagnoli E. (2004). Relationship of diabetes-specific knowledge to self-management activities, ambulatory preventive care, and metabolic outcomes. Prev. Med..

[36] Murata G.H., Shah J.H., Adam K.D., Wendel C.S., Bokhari S.U., Solvas P.A., Hoffman R.M., Duckworth W.C. (2003). Factors affecting diabetes knowledge in Type 2 diabetic veterans. Diabetologia.

[37] Al-Qazaz H., Sulaiman S.A., Hassali M.A., Shafie A.A., Sundram S., Al-Nuri R., Saleem F. (2011). Diabetes knowledge, medication adherence and glycemic control among patients with type 2 diabetes. Int. J. Clin. Pharm..

[38] Gunay T., Ulusel B., Velipasaoglu S., Unal B., Ucku R., Ozgener N. (2006). Factors affecting adult knowledge of diabetes in Narlidere Health District, Turkey. Acta Diabetol..

[39] Darr A., Astin F., Atkin K. (2008). Causal attributions, lifestyle change, and coronary heart disease: Illness beliefs of patients of South Asian and European origin living in the United Kingdom. Heart Lung.

[40] Fritz H., DiZazzo-Miller R., Bertran E.A., Pociask F.D., Tarakji S., Arnetz J., Lysack C.L., Jaber L.A. (2016). Diabetes self-management among Arab Americans: Patient and provider perspectives. BMC Int. Health Hum. Rights.

[41] Kanchanachitra C., Lindelow M., Johnston T., Hanvoravongchai P., Lorenzo F.M., Huong N.L. (2011). Human resources for health in southeast Asia: Shortages, distributional challenges, and international trade in health services. Lancet.

[42] Jin Y., Zhu W., Yuan B., Meng Q. (2017). Impact of health workforce availability on health care seeking behavior of patients with diabetes mellitus in China. Int. J. Equity Health.

[43] Brazeau A.S., Nakhla M., Wright M., Henderson M., Panagiotopoulos C., Pacaud D., Kearns P., Rahme E., Da Costa D., Dasgupta K. (2018). Stigma and Its Association with Glycemic Control and Hypoglycemia in Adolescents and Young Adults with Type 1 Diabetes: Cross-Sectional Study. J. Med. Internet Res..

[44] Abdoli S.A.P., Mardanian L. (2013). Exploring diabetes type 1-related stigma. Iran. J. Nurs. Midwifery Res..

[45] Abdoli S., Doosti I.M., Hardy L.R., Funnell M. (2018). A discussion paper on stigmatizing features of diabetes. Nurs. Open.

[46] Maslakpak H.M., Anoosheh A.M., Fazlollah A., Ebrahim H. (2010). Iranian diabetic adolescent girls’ quality of life: Perspectives on barriers. Scand. J. Caring Sci..

